# sFGL2 as a Potential Immunosuppressive Biomarker Associated With COVID‐19 Severity in Kidney Transplant Recipients

**DOI:** 10.1002/iid3.70296

**Published:** 2025-10-27

**Authors:** Yufei Zhang, Min Yang, Kai Liu, Jiang Zhu, Tianyin Wang, Peng Ding, Yingzi Ming, Bo Peng

**Affiliations:** ^1^ Transplantation Center, The Third Xiangya Hospital Central South University Changsha China; ^2^ NHC Key Laboratory of Translational Research on Transplantation Medicine Changsha China

**Keywords:** biomarker, COVID‐19, immunosuppression, kidney transplantation, sFGL2

## Abstract

**Background:**

SARS‐CoV‐2 infection can induce persistent immunosuppression. Soluble fibrinogen‐like protein 2 (sFGL2) is an emerging immune regulator. However, the correlation between sFGL2 and SARS‐CoV‐2–induced immunosuppression in kidney transplant recipients (KTRs) remains unclear.

**Materials and Methods:**

sFGL2 levels and peripheral blood lymphocyte subpopulations (PBLSs) were measured simultaneously in 50 KTRs with COVID‐19 on Day 1 and Day 7 after admission. An additional cohort of 15 stable KTRs without COVID‐19 was recruited as the control group. sFGL2 was quantified using enzyme‐linked immunosorbent assay, and PBLSs were analyzed with 6‐Color TBNK Reagent and quantified by flow cytometry.

**Results:**

sFGL2 levels in the COVID‐19 group were significantly higher than those in the stable group [64.33 ng/mL, interquartile range (IQR) 45.32–111.94 ng/mL vs. 53.82 ng/mL, IQR 31.31–72.63 ng/mL; *p* = 0.029]. Within the COVID‐19 group, KTRs with pneumonia exhibited markedly higher sFGL2 levels than those without pneumonia (97.29 ng/mL, IQR 74.13–141.82 ng/mL vs. 45.13 ng/mL, IQR 33.07–55.82 ng/mL; *p* < 0.001). sFGL2 correlated with disease severity (*r* = 0.692; *p* < 0.001), and sFGL2 > 70.58 ng/mL was identified as a risk factor for pneumonia [odds ratio (OR) 128.697; 95% confidence interval (CI) 8.339–1985.665; *p* < 0.001]. In addition, absolute PBLS counts were decreased in the COVID‐19 group, and CD3^+^ and CD8^+^ T‐cell counts were negatively correlated with sFGL2 (*r* = –0.241 and –0.278; *p* = 0.032 and 0.013, respectively). With clinical improvement of COVID‐19, sFGL2 levels decreased while PBLS counts recovered.

**Conclusion:**

sFGL2 was associated with immunosuppression, disease severity, and prognosis in KTRs with COVID‐19, suggesting that it could serve as a novel biomarker for monitoring immune status.

**Abbreviations**
AIH: autoimmune hepatitis
CI: confidence interval
CNI: calcineurin inhibitor
COVID‐19: coronavirus disease 2019
CRP: C‐reactive protein
DAMPs: damage‐associated molecular patterns
DCD: donation after citizen's death
Fc: fragment crystallizable
IL‐6: interleukin‐6
IQR: interquartile range
KTRs: kidney transplant recipients
MDSCs: myeloid‐derived suppressor cells.
OR: odds ratio
PBLSs: peripheral blood lymphocyte subpopulations
PLT: platelet count
ROC: receiver operating characteristic
Scr: serum creatinine
SD: standard deviation
sFGL2: soluble fibrinogen‐like protein 2
SOT: solid organ transplantation
Tregs: regulatory T cells
WBC: white blood cell

## 1 Introduction

The coronavirus disease 2019 (COVID‐19) pandemic has had a global negative impact on solid organ transplantation (SOT). According to statistical data, worldwide SOT decreased by 16% in 2020, with kidney transplantation being the most affected [1]. Although mortality related to COVID‐19 sharply declined after the introduction of vaccines and antiviral therapy in 2020 [2], immunocompromised patients—such as SOT recipients—experienced limited protection from vaccination [3]. Post–COVID‐19 SOT recipients exhibited a higher incidence of complications during hospitalization, with a hospital mortality rate 2.5 times greater than that of non‐transplanted patients [4]. Therefore, COVID‐19 remains a critical challenge for SOT recipients.

Fibrinogen‐like protein 2 (FGL2) is a member of the fibrinogen superfamily that exists in two forms: the membrane‐bound form, which induces immune‐associated coagulation, and the soluble form, which suppresses immune responses [5]. FGL2 is involved in modulating responses to tissue injury [6], malignancy [7], viral infection [8], acute allograft rejection [9], and autoimmune disease [10]. Soluble fibrinogen‐like protein 2 (sFGL2) is a novel effector produced by regulatory T cells (Tregs) that functions as an immunosuppressive regulator by binding to the low‐affinity fragment crystallizable (Fc) receptor FcγIIB. sFGL2 suppresses the maturation of dendritic cells, reduces costimulation of effector T cells, and induces apoptosis in B cells, thereby leading to an immunosuppressive state [11]. In addition, a multicenter study indicated that in COVID‐19 patients hospitalized for more than 24 h, FGL2 expression predicted the likelihood of respiratory bacterial superinfection [2]. Another study reported that FGL2 levels were higher in COVID‐19 patients who required mechanical ventilation and subsequently experienced fatal outcomes [12]. Furthermore, sFGL2 concentrations were significantly correlated with the development and mortality of sepsis in trauma patients [13]. Taken together, this evidence suggests that sFGL2 may be associated with COVID‐19–induced immunosuppression.

In this study, we measured sFGL2 levels in kidney transplant recipients (KTRs) with COVID‐19 and further investigated its clinical significance by analyzing correlations with disease severity and lymphopenia. This approach aimed to evaluate sFGL2 as a potential novel biomarker of immune dysregulation in this high‐risk population.

## 2 Methods

### 2.1 Study Design

From May 2023 to November 2023, 64 consecutive KTRs suspected of having COVID‐19 were recruited based on clinical presentation at the Third Xiangya Hospital, Central South University. One patient was subsequently excluded from the final analysis due to the absence of a laboratory‐confirmed SARS‐CoV‐2 nucleic acid test. Peripheral blood lymphocyte subpopulation (PBLS) testing was performed to assess immune function on day 1 and day 7 after admission. All participants were between 18 and 65 years of age. The exclusion criteria for COVID‐19 KTRs were as follows: (1) no PBLS test result, (2) history of re‐transplantation or multiple transplantation, or (3) combined multiple organ transplantation. Fifteen stable outpatient KTRs undergoing follow‐up were also recruited as the control group. The eligibility criteria for the stable KTRs were: (1) transplant time > 1 year, (2) no evidence of rejection, tumor, or infection, and (3) stable allograft function (serum creatinine < 171 μmol/L). All participants provided informed consent, and the study was approved by the Institutional Review Board of the Third Xiangya Hospital, Central South University (No. 23549).

All KTRs underwent kidney transplantation from donation after citizen's death (DCD) or from close family members after 2015. Each transplant was authorized by the Central South University Third Xiangya Hospital Ethics Committee. Donated organs were obtained with voluntary, informed consent, free of coercion, from the donor(s) or their next of kin, and no organs or tissues were procured from executed prisoners or prisoners of conscience.

### 2.2 Diagnostic Criteria of COVID‐19

COVID‐19 in KTRs was confirmed by SARS‐CoV‐2 nucleic acid testing. The *Diagnosis and Treatment Protocol* for SARS‐CoV‐2 *I*nfection (Trial Version 10) issued by the National Health Commission of China was applied to assess disease severity (Supporting Information S1: Table 1) [14]. Based on clinical manifestations, laboratory test results, radiological examinations, and this protocol, patients were classified into the pneumonia group and the non‐pneumonia group.

Basiliximab or anti‐human thymocyte immunoglobulin was used as induction therapy for kidney transplantation. The maintenance immunosuppressive regimen consisted of calcineurin inhibitors (CNIs), mycophenolate mofetil (MMF), and corticosteroids. All COVID‐19 patients received antiviral therapy as clinically indicated during hospitalization. In principle, all KTRs diagnosed with COVID‐19 were required to discontinue mycophenolate mofetil or enteric‐coated mycophenolate sodium. Patients treated with molnupiravir continued to receive CNIs and corticosteroids, whereas those treated with paxlovid discontinued CNIs but continued corticosteroids. Immunosuppressant dosages were adjusted according to serum creatinine levels and drug concentrations.

### 2.3 sFGL2 Assay

Plasma for sFGL2 detection was collected from the same blood samples used for PBLS testing and stored at –80°C until analysis. sFGL2 was measured with a commercially available enzyme‐linked immunosorbent assay (ELISA; BioLegend, San Diego, CA, USA) according to the manufacturer's instructions.

### 2.4 PBLS Test

The BD Multitest 6‐Color TBNK Reagent with BD Trucount tubes was used to determine the percentages and absolute counts of CD3^+^CD4^+^ T cells, CD3^+^CD8^+^ T cells, CD19^+^ B cells, and natural killer (NK) cells. Blood samples in BD Trucount tubes were incubated with mixed antibodies for 15 min at room temperature in the dark. After erythrolysis, the samples were analyzed using a BD FACSCanto II flow cytometer, and the data were processed with BD FACSCanto Clinical Software (BD Biosciences, San Jose, CA, USA).

### 2.5 Statistical Analysis

Continuous variables were expressed as mean ± standard deviation (SD) or median with interquartile range (IQR) and compared using Student's *t* test, Welch's *t* test, or Mann–Whitney *U* test, as appropriate. Categorical variables were analyzed using the *χ* ^2^ test or Fisher's exact test, as appropriate. Correlations were assessed using Spearman's correlation analysis. The cut‐off value of sFGL2 was determined by receiver operating characteristic (ROC) curve analysis. Logistic regression analysis was used to identify risk factors. Statistical analyses were performed using SPSS version 26.0 (SPSS, Inc., Chicago, IL, USA) and GraphPad Prism 10.0. *p* values < 0.05 were considered statistically significant.

## 3 Results

### 3.1 Patient Characteristics

**Figure 1:**
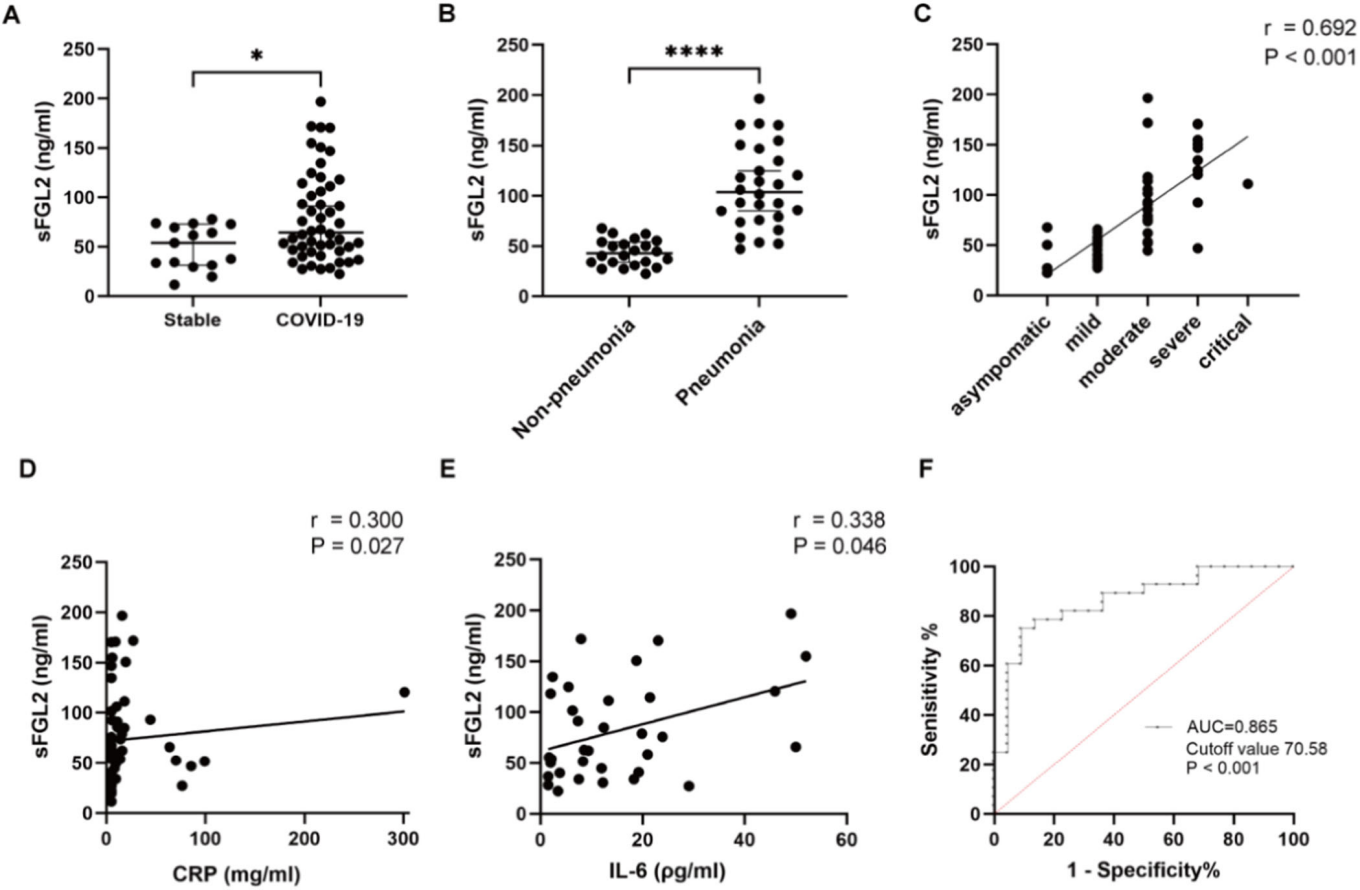
Levels of sFGL2 and correlations with COVID‐19 severity. (A) sFGL2 levels in the COVID‐19 group (*n* = 50) vs. the stable group (*n* = 15), tested using the Mann–Whitney *U* test. (B) sFGL2 levels in the pneumonia subgroup (*n* = 22) vs. the non‐pneumonia subgroup (*n* = 28), tested using the Mann–Whitney *U* test. (C) Correlation between sFGL2 levels and COVID‐19 severity (*n* = 50), tested using Spearman's rank correlation coefficient. (D) Correlation between sFGL2 and CRP (*n* = 53), tested using Spearman's rank correlation coefficient. (E) Correlation between sFGL2 and IL‐6 (*n* = 35), tested using Spearman's rank correlation coefficient. (F) ROC curve of sFGL2 for diagnosing COVID‐19 pneumonia, with cut‐off values determined by the Youden index. AUC, area under the curve; CRP, C‐reactive protein; IL‐6, interleukin‐6; ROC, receiver operating characteristic; sFGL2, soluble fibrinogen‐like protein 2. Significance levels: *p < 0.05, **p < 0.01, ***p < 0.001, ****p < 0.0001; ns, not significant.

Of the 63 KTRs with confirmed or highly probable COVID‐19, 50 were confirmed by SARS‐CoV‐2 nucleic acid testing and were included in the final analysis. Based on diagnostic criteria, 22 KTRs were classified into the non‐pneumonia subgroup (18 mild cases and 4 asymptomatic cases), and the remaining 28 were classified into the pneumonia subgroup (1 critical case, 10 severe cases, and 17 moderate cases). A total of 64 measurements of sFGL2 and PBLSs were available for analysis. All patients underwent the first assessment on day 1 after admission. Due to short hospital stays and rapid treatment, only 14 patients underwent a second assessment. The study flow is presented in Supporting Information S1: Figure 1.

Patient characteristics are summarized in Table 1. There were no significant differences in age, sex, transplant time, CNI regimen, donor source, white blood cell (WBC) count, serum creatinine (Scr), or platelet count (PLT) between the COVID‐19 and stable groups or between the pneumonia and non‐pneumonia subgroups. In our center, 60% of COVID‐19 KTRs reduced or discontinued CNIs. Compared with the non‐pneumonia group, the frequency of CNI dose reduction or discontinuation was higher in the pneumonia group; however, the difference was not statistically significant. PBLS testing was performed concurrently with monitoring of CNI concentration. At admission, there was no significant difference in tacrolimus concentration between COVID‐19 KTRs and the stable group (6.10 ng/mL, IQR 4.30–7.50 ng/mL vs. 6.20 ng/mL, IQR 5.70–7.20 ng/mL; *p* = 0.063). Compared with the non‐pneumonia group, tacrolimus concentration in the pneumonia group was slightly higher but not significantly different (6.50 ng/mL, IQR 5.57–8.12 ng/mL vs. 5.70 ng/mL, IQR 3.25–7.35 ng/mL; *p* = 0.089). The COVID‐19 group, however, had significantly reduced lymphocyte counts, particularly in T‐ and B‐cell populations, compared with the stable group. Within the COVID‐19 group, the pneumonia subgroup had lower T‐cell counts but higher levels of inflammatory factors, including C‐reactive protein (CRP) and interleukin‐6 (IL‐6). The time to nucleic acid negativity was also longer in the pneumonia subgroup. No patient died during treatment, and only one patient in the pneumonia subgroup lost the allograft.

**Table 1:** Clinical characteristics of COVID‐19 KTRs.

		COVID‐19 (*n* = 50)					
		Non‐pneumonia	Pneumonia				
Variable	COVID‐19 (*n* = 50)	(*n* = 22)	(*n* = 28)		*p* value^b^	Stable (*n* = 15)	*p* value^a^
Age^f^(years, mean ± SD)	46.50 ± 10.20	44.54 ± 11.52	48.07 ± 9.11		0.724	45.50 ± 8.20	0.606
Male, *n* (%)^d^	30 (60.0%)	12 (54.5%)	18 (64.3%)		0.485	10 (66.7%)	0.642
Transplant time^e^, (months, median [IQR])	44.0	51.0	36.5		0.667	61.0	0.994
	(17.5, 99.0)	(18.8, 78.0)	(4.7, 36.5)			(14.0, 81.0)	
Donor^c^, *n* (%)					0.616		0.582
DCD	45 (90.0%)	20 (90.9%)	25 (89.3%)			15 (100%)	
Relative	5 (10.0%)	2 (9.1%)	3 (10.7%)			0 (0%)	
Antiviral therapy^d^, *n* (%)					0.515		NA
None	8 (16.0%)	5 (22.7%)	3 (10.7%)			_	
Paxlovid	20 (40.0%)	8 (36.4%)	12 (42.9%)				
Molnupiravir	22 (44.0%)	9 (40.9%)	13 (46.4%)				
Immunosuppression							
Maintenance at admission^c^, *n* (%)					0.833		0.528
FK506	46 (92.0%)	20 (90.9%)	26 (92.9%)			15 (100.0%)	
CsA	3 (6.0%)	2 (7.1%)	1 (4.5%)			0 (0%)	
FK506 + Siro	1 (1.9%)	0 (0.0%)	1 (4.5%)			0 (0%)	
Adjustment after admission, *n* (%)							
FK506 reduction/discontinuation	30 (60.0%)	10 (45.5%)	20 (71.4%)		0.063	0 (0%)	0.003
MMF reduction/discontinuation	32 (64.0%)	14 (63.6%)	18 (64.3%)		0.962	1 (6.7%)	< 0.001
Steroid reduction/discontinuation	22 (44.0%)	8 (36.4%)	14 (50.0%)		0.335	0 (0.0%)	0.002
FK506 (ng/mL, median [IQR])	6.1 (4.3, 7.5)	6.5 (5.57,8.1)	5.7 (3.25,7.3)		0.089	6.2 (5.7, 7.2)	0.063
sFGL2^e^, (ng/mL, median [IQR])	64.33	45.13	97.29		< 0.001	53.82	0.029
	(45.32, 111.94)	(33.07, 55.82)	(74.13, 141.82)			(31.31, 72.63)	
CD3^+^ T cells^e^, (cells/μL, median [IQR])	579	688	546		0.037	1233	< 0.001
	(424, 804)	(468, 1309)	(385, 713)			(1143, 1552)	
CD8^+^ T cells^e^, (cells/μL, median [IQR])	263	288	245		0.022	518	< 0.001
	(185, 338)	(202, 517)	(168, 310)			(424, 631)	
CD4^+^ T cells^e^, (cells/μL, median [IQR])	315	361	265		0.036	683	< 0.001
	(194, 388)	(175, 676)	(202, 340)			(495, 966)	
B cells^e^, (cells/μL, median [IQR])	76	86	70		0.177	143	0.004
	(30, 141)	(37, 158)	(21, 100)			(98, 217)	
NK cells^e^, (cells/μL, median [IQR])	136	162	131		0.282	135	0.767
	(78, 246)	(66, 296)	(82, 187)			(86, 217)	
TBNK^e^, (cells/μL, median [IQR])	883	1029	804		0.055	1653	< 0.001
	(544, 1111)	(572, 1830)	(466, 1051)			(1357, 1964)	
WBC e, (×10^9^/L, median [IQR])	5.07	4.94	5.43		0.374	6.73	0.63
	(3.91, 6.83)	(3.51, 6.79)	(4.09, 6.77)			(4.63, 7.42)	
Lymphocytes^e^, (×10^9^/L, median [IQR])	0.88	1.02	0.87		0.051	1.66	< 0.001
	(0.76, 1.23)	(0.77, 1.83)	(0.58, 1.01)			(1.45, 1.90)	
Platelets^f^, (×10^9^/L, mean ± SD)	162 ± 79	163 ± 46	168 ± 62		0.734	189 ± 39	0.068
Total bilirubin^e^, (μmol/L, median [IQR])	8.9	9.5	7.9		0.358	12.6	0.006
	(6.4, 10.6)	(6.5, 11.5)	(6.1, 9.5)			(8.9, 15.7)	
Scr^e^, (μmol/L, median [IQR])	106	90	112		0.233	97	0.084
	(83, 154)	(70, 161)	(91, 136)		97	(71, 107)	
CRP^e^, (mg/L, median [IQR])	10.28	5.08	10.61		0.020	_	NA
	(5.16, 17.93)	(5.00, 7.76)	(5.00, 19.24)				
IL‐6^e^, (pg/mL, median [IQR])	10.65	7.47	19		0.005	_	NA
	(3.40, 21.80)	(2.01, 12.09)	(6.77, 47.48)				
Time to negative nucleic acid^f^(days, mean ± SD)	11.2 ± 5.5	8.4 ± 3.5	13.5 ± 5.9		< 0.001	_	NA
Graft loss, *n* (%)	1 (2.0%)	0 (0.0%)	1 (3.6%)		0.371	_	NA

Abbreviations: AKI, acute kidney injury; B cells, absolute B‐lymphocyte count; CD3^+^ T, absolute CD3+ T‐lymphocyte count; CD4^+^ T, absolute CD4^+^ T‐lymphocyte count; CD8^+^ T, absolute CD8^+^ T‐lymphocyte count; CNI, calcineurin inhibitor; CRP, C‐reactive protein; DCD, donation after citizen's death; IL‐6, interleukin‐6; LYM, lymphocyte; NK cells, absolute NK‐cell count; Plt, platelet count; Scr, serum creatinine; sFGL2, soluble fibrinogen‐like protein 2; Siro, sirolimus; Tbil, total bilirubin; TBNK, absolute lymphocyte count of T, B, and NK cells; WBC, white blood cell.

^a^ Comparison between pneumonia patients and stable patients.

^b^ Comparison between pneumonia patients and non‐pneumonia patients.

^c^ Tested using Fisher's exact test.

^d^ Tested using the *χ* ^2^ test.

^e^ Tested using the Mann–Whitney *U* test.

f Tested using Welch's *t* test.

### 3.2 sFGL2 Was Increased in COVID‐19 KTRs and Correlated With the Disease Severity

sFGL2 levels were significantly higher in the COVID‐19 group than in the stable group (64.33 ng/mL, IQR 45.32–111.94 ng/mL vs. 53.82 ng/mL, IQR 33.31–72.63 ng/mL; *p* = 0.029; Figure 1A). Within the COVID‐19 group, the pneumonia subgroup also showed elevated sFGL2 levels compared with the non‐pneumonia subgroup (97.29 ng/mL, IQR 74.13–141.82 ng/mL vs. 45.13 ng/mL, IQR 33.07–55.82 ng/mL; *p* < 0.001; Figure 1B). Correlation analysis confirmed a positive association between COVID‐19 disease severity and sFGL2 (*r* = 0.692; *p* < 0.001; Figure 1C). Additionally, sFGL2 was positively correlated with CRP (*r* = 0.300; *p* = 0.027; Figure 1D) and with IL‐6 (*r* = 0.338; *p* = 0.046; Figure 1E).

ROC curve analysis was subsequently performed to assess the ability of sFGL2 to distinguish between pneumonia and non‐pneumonia COVID‐19 KTRs. The area under the curve (AUC) was 0.865 (*p* < 0.001; Figure 1F). Using a cut‐off value of 70.58 ng/mL determined by the optimal Youden index, sensitivity was 0.750 and specificity was 0.909.

To further explore the relationship between sFGL2 and COVID‐19, univariate logistic regression analysis was performed to identify risk factors associated with pneumonia in COVID‐19 KTRs. Variables considered clinically relevant and showing significant differences were included in the univariate analysis (Table 2). The results showed that sFGL2 > 70.58 ng/ml, as well as cell counts of CD3^+^ T cells, CD8^+^ T cells, CD4^+^ T cells, and lymphocytes, were risk factors for pneumonia in COVID‐19 KTRs. Variables with *p* values < 0.15 were subsequently included in multivariate analysis. The results revealed that sFGL2 > 70.58 ng/mL was an independent risk factor [odds ratio (OR) 128.697; 95% confidence interval (CI) 8.339–1985.665; *p* < 0.001].

**Table 2:** Univariate and multivariate odds ratios for pneumonia diagnosis among COVID‐19 KTRs.

Parameters	Univariate analysis			Multivariate analysis		
	OR	95% (CI)	*p*‐value	OR	95% (CI)	*p* value
Age (years)	1.048	(0.981, 1.119)	0.164			
Gender	1.832	(0.530, 6.335)	0.339			
sFGL2 > 70.58 ng/mL	77.000	(8.533, 694.806)	< 0.001	128.697	(8.339, 1985.665)	< 0.001
CD3^+^ T cells (cells/μL)	0.998	(0.996, 1.000)	0.022	0.988	(0.958, 1.018)	0.430
CD8^+^ T cells (cells/μL)	0.995	(0.990, 1.000)	0.031	1.007	(0.972, 1.043)	0.711
CD4^+^ T cells (cells/μL)	0.996	(0.993, 1.000)	0.038	1.006	(0.974, 1.039)	0.728
B cells (cells/μL)	0.999	(0.992, 1.005)	0.658			
NK cells (cells/μL)	0.998	(0.994, 1.001)	0.213			
CRP (mg/L)	1.007	(0.990, 1.024)	0.425			
Lymphocytes (×10^9^/L)	0.257	(0.074, 0.895)	0.033	4.377	(0.025, 762.929)	0.575

Abbreviations: B cells, absolute B‐lymphocyte count; CI, confidence interval; CD3^+^ T, absolute CD3^+^ T‐lymphocyte count; CD4^+^ T, absolute CD4^+^ T‐lymphocyte count; CD8^+^ T, absolute CD8^+^ T‐lymphocyte count; CRP, C‐reactive protein; IL‐6, interleukin‐6; NK cells, absolute NK‐cell count; Lymphocytes, total lymphocyte count; OR, odds ratio; sFGL2, soluble fibrinogen‐like protein 2.

### 3.3 sFGL2 Correlated With Decreased CD8^+^ T Cells in COVID‐19 KTRs

One of the most significant features of COVID‐19 was immunosuppression, which manifested as lymphopenia. As shown in Table 1, COVID‐19 KTRs had significantly lower lymphocyte counts than stable controls. Specifically, for lymphocyte subpopulations, counts of CD3^+^ T cells [579/μL, IQR 424–804/μL vs. 1233/μL, IQR 1143–1552/μL; *p* < 0.001], CD8^+^ T cells [263/μL, IQR 185–338/μL vs. 518/μL, IQR 424–631/μL; *p* < 0.001], CD4^+^ T cells [315/μL, IQR 194–388/μL vs. 683/μL, IQR 495–966/μL; *p* < 0.001], and B cells [76/μL, IQR 30–141/μL vs. 143/μL, IQR 98–217/μL; *p* = 0.004] were lower in the COVID‐19 group than in the stable group, whereas NK‐cell counts [136/μL, IQR 78–246/μL vs. 135/μL, IQR 86–217/μL; *p* = 0.767] showed no significant difference (Table 1). Within the COVID‐19 group, the pneumonia subgroup mainly exhibited a significant decrease in T‐cell counts compared with the non‐pneumonia subgroup.

**Figure 2:**
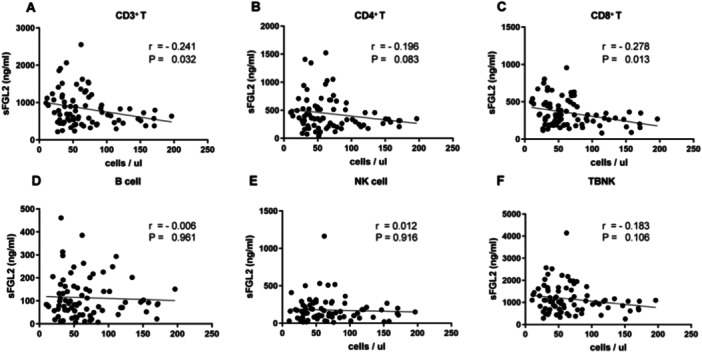
Correlation between sFGL2 and peripheral blood lymphocyte subpopulations (*n* = 79). Each correlation was tested using Spearman's rank correlation coefficient. (A) sFGL2 and CD3^+^ T cells. (B) sFGL2 and CD4^+^ T cells. (C) sFGL2 and CD8^+^ T cells. (D) sFGL2 and B cells. (E) sFGL2 and NK cells. (F) sFGL2 and TBNK. sFGL2, soluble fibrinogen‐like protein 2; TBNK, absolute lymphocyte counts of T, B, and NK cells.

To further investigate the role of sFGL2 in immunosuppression, correlation analysis was performed between sFGL2 and PBLSs (Figure 2). The results indicated that sFGL2 was negatively correlated with CD3^+^ T‐cell counts (*r* = –0.241; *p* = 0.032) and CD8^+^ T‐cell counts (*r* = –0.278; *p* = 0.013). These findings suggest that sFGL2 may be primarily associated with decreased CD8^+^ T cells in COVID‐19.

### 3.4 Longitudinal Changes of sFGL2 and Prognosis in COVID‐19 KTRs

**Figure 3:**
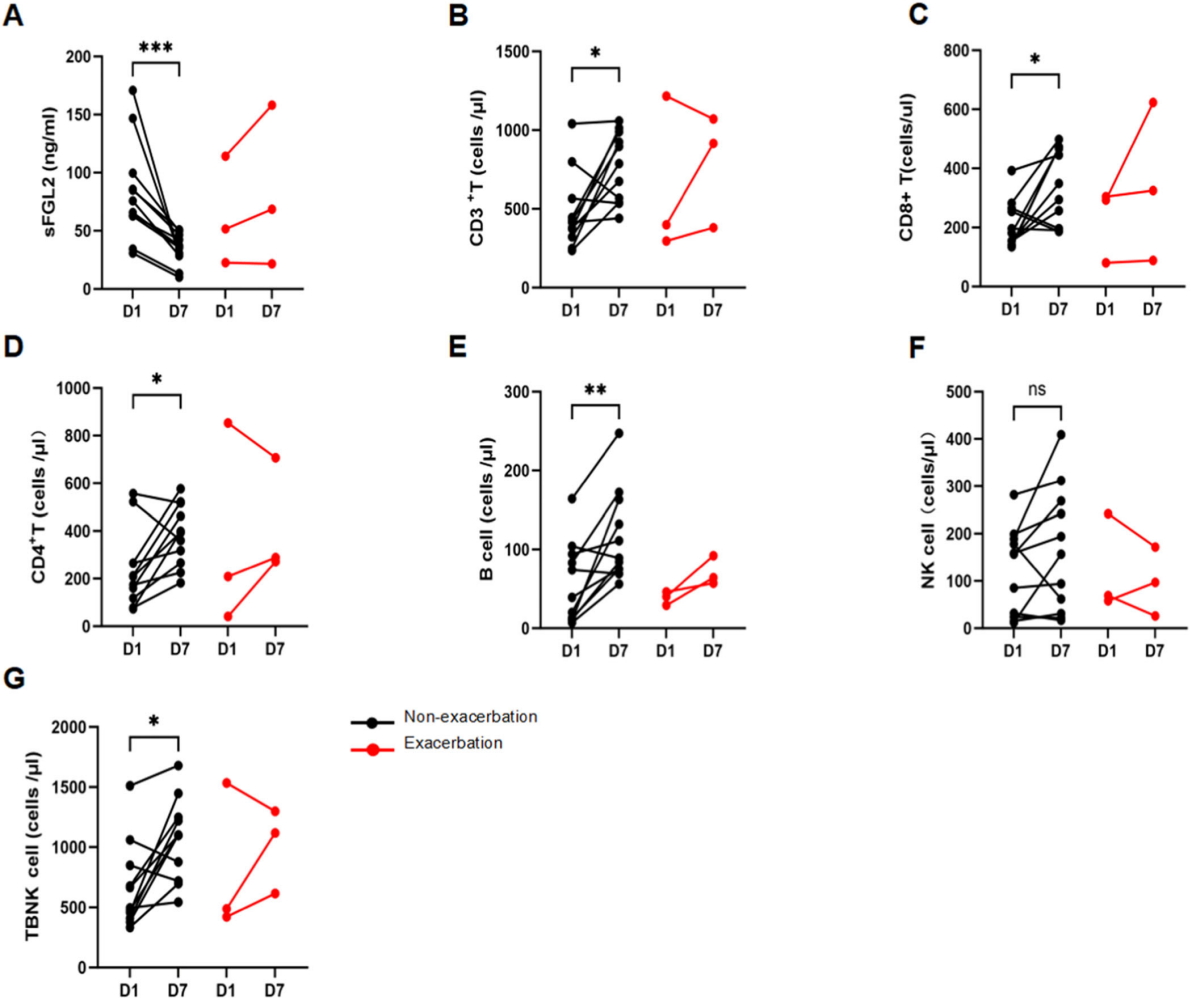
Longitudinal changes in sFGL2 and peripheral blood lymphocyte subpopulation counts in COVID‐19 KTRs. Data from day 1 and day 7 were compared in the exacerbation subgroup (*n* = 3) and the non‐exacerbation subgroup (*n* = 11). Subgroups were defined according to the Diagnosis and Treatment Protocol for SARS‐CoV‐2 Infection (Trial Version 10*)* issued by the National Health Commission of China. The non‐exacerbation subgroup was analyzed using the paired t test. The exacerbation subgroup was not subjected to statistical analysis due to the small sample size (*n* = 3); only individual trends are shown. (A) sFGL2 levels. (B) Absolute CD3^+^ T‐cell counts. (C) Absolute CD4^+^ T‐cell counts. (D) Absolute CD8^+^ T‐cell counts. (E) Absolute B‐cell counts. (F) Absolute NK‐cell counts. (G) Absolute TBNK counts. sFGL2, soluble fibrinogen‐like protein 2; TBNK, absolute lymphocyte counts of T, B, and NK cells. Significance levels: *p < 0.05, **p < 0.01, ***p < 0.001, ****p < 0.0001; ns, not significant.

A total of 14 patients underwent both sFGL2 and PBLS testing on day 1 and day 7 after admission. According to COVID‐19 disease severity, 3 patients experienced exacerbation and 11 patients improved or maintained their original status. Longitudinal comparisons showed a significant decrease in sFGL2 levels in the non‐exacerbation subgroup (75.72 ng/mL, IQR 62.83–99.49 ng/mL vs. 35.44 ng/mL, IQR 28.61–46.34 ng/mL; *p* = 0.002), whereas the exacerbation subgroup showed a trend toward increased levels (Figure 3A). In the non‐exacerbation subgroup, lymphocyte subpopulation counts significantly increased, including CD3^+^ T cells [416/μL, IQR 322–565/μL vs. 787/μL, IQR 540–989/μL; *p* = 0.010], CD8^+^ T cells [181/μL, IQR 153–265/μL vs. 349/μL, IQR 196–470/μL; *p* = 0.012], CD4^+^ T cells [209/μL, IQR 118–266/μL vs. 394/μL, IQR 266–571/μL; *p* = 0.014], and B cells [39/μL, IQR 11–94/μL vs. 89/μL, IQR 75–163/μL; *p* = 0.003], while NK‐cell counts [157/μL, IQR 24–189/μL vs. 157/μL, IQR 31–269/μL; *p* = 0.137] showed no significant difference. In contrast, lymphocyte subpopulation counts in the exacerbation subgroup only demonstrated individual trends, which could not be statistically confirmed due to the small sample size.

## 4 Discussion

While COVID‐19 management has shifted toward addressing long‐term sequelae, immunocompromised populations such as KTRs remain vulnerable to severe outcomes [15]. Lumlertgul et al. reported that the incidence of lymphopenia was as high as 90.0% in 393 patients with COVID‐19 [16]. Furthermore, lymphopenia has been shown to be associated with both disease severity and mortality in COVID‐19 [17]. Prolonged viral exposure in immunodeficient hosts may also promote mutational evolution [18]. Our retrospective study demonstrated that sFGL2 levels were significantly elevated in KTRs with COVID‐19 compared with stable controls. Further analysis revealed a positive correlation between elevated sFGL2 levels and disease severity, as well as a negative correlation with CD8^+^ T‐cell counts. These findings highlight the importance of exploring novel biomarkers associated with immune function and prognosis in COVID‐19 KTRs.

Persistent inflammation and immunodeficiency act synergistically in the progression of COVID‐19 [19]. During infection, SARS‐CoV‐2 activates damage‐associated molecular patterns (DAMPs), leading to hyperinflammation characterized by a “cytokine storm” in severe cases [20, 21]. Pro‐inflammatory cytokines, including IL‐1β, IL‐6, and tumor necrosis factor‐alpha, are markedly upregulated, causing uncontrolled inflammation and multiorgan damage. At the same time, hyperinflammation results in a substantial loss of T cells and restricts T‐cell proliferation under persistent inflammatory conditions [22]. Severe and critical COVID‐19 patients also demonstrate reduced clearance efficiency of SARS‐CoV‐2 due to sustained inflammatory responses, increased frequencies of circulating Treg cells, severe lymphopenia, and T‐cell functional exhaustion [23]. In KTRs, impaired clearance of SARS‐CoV‐2 predisposes to viral persistence and recurrent infections, further aggravating immune dysfunction and T‐cell exhaustion after COVID‐19. Other studies have reported that patients with COVID‐19 exhibit lymphopenia accompanied by a significant reduction in T‐cell subsets, with CD4^+^ and CD8^+^ T‐cell numbers progressively declining as the disease advances [24]. Our results confirmed lymphopenia in COVID‐19 KTRs: CD3^+^ T cells, CD8^+^ T cells, CD4^+^ T cells, and B cells were decreased during infection. However, in the pneumonia subgroup, only T cells and their subsets were reduced, which was accompanied by elevated levels of the pro‐inflammatory factors CRP and IL‐6. These results indicate that immune dysregulation—manifesting as both hyperinflammation and immunosuppression—was present in COVID‐19 KTRs, particularly in severe cases.

In previous studies, sFGL2 was shown to exert both pro‐inflammatory and immunomodulatory effects, similar to the pathological mechanisms observed in COVID‐19. This suggests that sFGL2 may play a role in COVID‐19 progression. In COVID‐19, FGL2 has been found to be closely associated with TIGIT^+^ Tregs. De Lima et al. observed that TIGIT^+^ Tregs were elevated in COVID‐19 patients requiring mechanical ventilation, accompanied by increased FGL2 expression [12]. TIGIT on Tregs can induce FGL2 expression, which in turn promotes cell‐mediated inhibition of effector T‐cell proliferation [25]. In our study, we confirmed elevated sFGL2 levels in COVID‐19 KTRs. Furthermore, sFGL2 levels were higher in the pneumonia subgroup and correlated with disease severity. KTRs with high sFGL2 levels were more likely to develop pneumonia during COVID‐19. When COVID‐19 improved, sFGL2 levels declined. These findings support a role for sFGL2 in the progression of COVID‐19 in KTRs.

Immunosuppression has recently been highlighted during COVID‐19, with impaired T cell–mediated immunity identified as an important factor in the development of severe disease. Both our results and previous reports support that patients with severe COVID‐19 exhibit decreased T cells, particularly effector CD8^+^ T cells [26]. As an immunosuppressive effector, sFGL2 produced by Tregs can impede the progression of autoimmune hepatitis (AIH) by inhibiting conventional CD8^+^ T cells [27]. sFGL2 has also been shown to promote tumor progression through regulation of cholesterol metabolism in myeloid‐derived suppressor cells (MDSCs), with the key mechanism being suppression of CD8^+^ T‐cell proliferation [28]. In addition, sFGL2 can directly bind to FcγRIIB on CD8^+^ effector T cells and induce apoptosis via caspase‐3/7 activation [29]. Anna B. Morris et al. reported that both chronically LCMV‐infected mice and COVID‐19 patients exhibit significant increases in plasma Fgl2 levels. Exposure of CD8^+^ T cells to an Fgl2‐enriched environment led to depletion of FcγRIIB^+^ CD8^+^ T cells. Furthermore, plasma Fgl2 levels in COVID‐19 patients were positively correlated with CD8^+^ T‐cell lymphopenia. Collectively, these findings indicate that during viral infection, Fgl2 produced by CD8^+^ T cells contributes to FcγRIIB‐mediated loss of immune function in CD8^+^ T cells in both murine models and humans [30]. This negative correlation suggests that elevated sFGL2 may contribute to CD8^+^ T‐cell depletion and thereby impair antiviral immunity in KTRs. Moreover, dynamic changes in sFGL2 levels were observed to coincide with the recovery of CD8^+^ T‐cell counts, further supporting the hypothesis that sFGL2 suppresses T‐cell activity by inducing apoptosis. These findings demonstrate the immunosuppressive capacity of sFGL2, which may contribute to impaired T cell–mediated immunity.

This study has several limitations. Patients were recruited from a single institution, and the sample size was relatively small. Only two sFGL2 measurements were performed within a 2‐week period for longitudinal observation, and most patients were discharged before completing all scheduled testing due to prompt therapy and rapid recovery. The relatively short detection window for sFGL2 limited the ability to evaluate its potential association with long‐term sequelae. Therefore, further studies are required to investigate the prognostic significance of sFGL2 in COVID‐19.

## 5 Conclusion

In summary, sFGL2 levels were significantly increased in COVID‐19 KTRs and correlated with disease severity and prognosis. sFGL2 also reflected the degree of immunosuppression and may serve as a novel immunosurveillance biomarker.

## Author Contributions

**Yufei Zhang:** methodology, resources, investigation, data curation, visualization, formal analysis, writing – original draft. **Min Yang:** methodology, resources, data curation. **kai liu:** resources, data curation. **Jiang Zhu:** resources, data curation. **Tianyin Wang:** resources, data curation. **Peng Ding:** resources, data curation. **Yingzi Ming:** writing – review and editing, supervision, project administration. **Bo Peng:** conceptualization, methodology, funding acquisition, writing – review and editing, supervision, project administration.

## Consent

All authors provided consent for publication.

## Conflicts of Interest

The authors declare no conflicts of interest.

## Supporting information


**Table S1:** The diagnostic criteria and classifications of COVID‐19. **Figure S1:** The study flow and diagnostic criteria of COVID‐19. 63 suspected COVID‐19 KTRs with 83 sFGL2 tests were enrolled, 13 patients with 19 tests were excluded according to the diagnostic criteria of COVID‐19. The remaining 50 patients were further classified into the pneumonia subgroup (*n* = 28) and non‐pneumonia subgroup (*n* = 22) according to the National Health Commission of China‐Diagnosis and Treatment Protocol for SARS‐CoV‐2 Infection (Trial Version 10). 14 of 50 patients recived the sceond test and grouped into exacerbation subgroup (*n* = 3) and non‐exacerbation subgroup (*n* = 11). sFGL2, soluble fibrinogen protein.

STROBE Statement—Checklist of items that should be included in reports of ***cross‐sectional studies***.

## Data Availability

The data that support the findings of this study are available on request from the corresponding author, upon reasonable request.
