# Supplementary material for: sFGL2 as a Potential Immunosuppressive Biomarker Associated With COVID‐19 Severity in Kidney Transplant Recipients
Source: Immun Inflamm Dis. 2025 Oct 27;13(10):e70296. doi: 10.1002/iid3.70296 (PMC12558890; doi:10.1002/iid3.70296)
Supplement: Supplementary file 1 — Table S1: The diagnostic criteria and classifications of COVID‐19. Figure S1: The study flow and diagnostic criteria of COVID‐19. 63 suspected COVID‐19 KTRs with 83 sFGL2 tests were enrolled, 13 patients with 19 tests were excluded according to the diagnostic criteria of COVID‐19. The remaining 50 patients were further classified into the pneumonia subgroup (n = 28) and non‐pneumonia subgroup (n = 22) according to the National Health Commission of China‐Diagnosis and Treatment Protocol for SARS‐CoV‐2 Infection (Trial Version 10). 14 of 50 patients recived the sceond test and grouped into exacerbation subgroup (n = 3) and non‐exacerbation subgroup (n = 11). sFGL2, soluble fibrinogen protein. [file IID3-13-e70296-s001.docx]

## **sFGL2 as a novel immunosuppressive biomarker in kidney transplant recipients with COVID-19**

**Supplementary Material**

**Contains:**

**Table S1. The diagnostic criteria and classifications of COVID-19**

**Figure S1. The study flow and diagnostic criteria of COVID-19.**

**Table S1. The diagnostic criteria and classifications of COVID-19**

| Classifications | Clinical manifestations and diagnostic criteria |
| --- | --- |
| Asymptomatic | 1. Only positive laboratory nucleic acid test for SARS-CoV-2 without other clinical manifestations. |
| Mild | People with positive nucleic acid test for SARS-CoV-2 meet the following conditions:   1. Upper respiratory tract infection as the main manifestation, including dry throat, sore throat, cough, fever. |
| Moderate | People with positive laboratory nucleic acid test for SARS-CoV-2 meet all the following conditions:  a. Persistent high fever > 3 days or (and) cough;  b. Shortness of breath, RR < 30 breaths/min;  c. SpO_2_ > 93% on air inhalation at rest;  d. Characteristic COVID-19 pneumonic imaging. |
| Severe | Adults with positive laboratory nucleic acid test for SARS-CoV-2 meet any of the following conditions, which cannot be explained by other reasons except the COVID-19:   1. Shortness of breath, RR  ≥ 30 breaths/min; 2. SpO_2_ is ≤ 93% on air inhalation at rest; 3. PaO_2_ /FiO_2_ ≤ 300 mmHg (1 mmHg = 0.133 kPa);   In areas with high altitude > 1000 m above the sea level, PaO_2_/FiO_2_ should be adjusted according to the formula: PaO_2_/FiO_2_ × [760/atmospheric pressure (mmHg)].   1. Lung imaging shows that the lesion has progressed significantly > 50% within 24 to 48 h. |
| Critical | People with positive laboratory nucleic acid test for SARS-CoV-2 meet any one of the following conditions:   1. Respiratory failure and mechanical ventilation; 2. Shock; 3. ICU admission due to organ failures. |

*PaO_2_, arterial partial pressure of oxygen; FiO_2_, the fraction of inspired oxygen; SpO_2_, the pulse oxygen saturation; RR,respiratory rate; ICU, intensive care unit.*


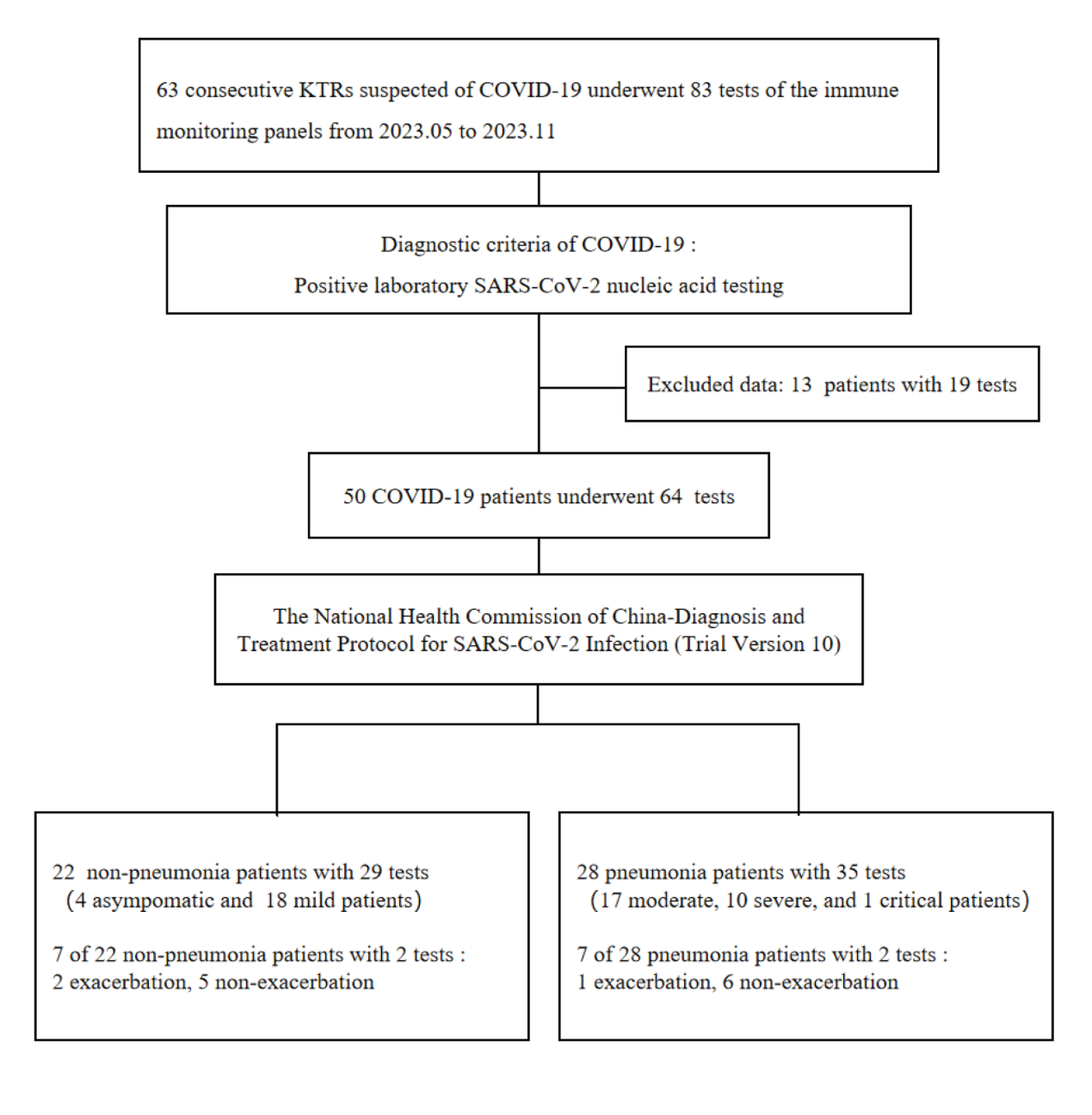


**Figure S1.** The study flow and diagnostic criteria of COVID-19. 63 suspected COVID-19 KTRs with 83 sFGL2 tests were enrolled, 13 patients with 19 tests were excluded according to the diagnostic criteria of COVID-19. The remaining 50 patients were further classified into the pneumonia subgroup (n = 28) and non-pneumonia subgroup (n = 22) according to the National Health Commission of China-Diagnosis and Treatment Protocol for SARS-CoV-2 Infection (Trial Version 10). 14 of 50 patients recived the sceond test and grouped into exacerbation subgroup (n = 3) and non-exacerbation subgroup (n = 11). sFGL2, soluble fibrinogen protein.
